# Early response of right-ventricular function to percutaneous mitral valve repair

**DOI:** 10.1007/s00392-021-01951-7

**Published:** 2021-10-20

**Authors:** Atsushi Sugiura, Jasmin Shamekhi, Tadahiro Goto, Maximilian Spieker, Christos Iliadis, Refik Kavsur, Victor Mauri, Malte Kelm, Stephan Baldus, Tetsu Tanaka, Noriaki Tabata, Jan-Malte Sinning, Marcel Weber, Sebastian Zimmer, Georg Nickenig, Ralf Westenfeld, Roman Pfister, Marc Ulrich Becher, of the Heart Failure Network Rhineland

**Affiliations:** 1 Department of Medicine II, Heart Center Bonn, University Hospital Bonn, Venusberg-Campus 1, 53127 Bonn, Germany; 2 Department of Clinical Epidemiology and Health Economics, School of Public Health, University of Tokyo, Tokyo, Japan; 3 Department of Cardiology, Heart Center, University Hospital Düsseldorf, Düsseldorf, Germany; 4 Department of Cardiology, Heart Center, University Hospital Cologne, Cologne, Germany; 5 Department of Cardiovascular Medicine, Graduate School of Medical Sciences, Kumamoto University, Kumamoto, Japan; 6 Department of Cardiology, St. Vinzenz-Hospital Cologne, Cologne, Germany

**Keywords:** Transcatheter mitral valve repair, MitraClip, Right ventricular function, Echocardiography, Heart failure, Prognosis

## Abstract

**Background:**

The change in right-ventricular function (RVF) after transcatheter mitral valve repair is still poorly understood. We assessed the early response of RVF to the MitraClip procedure and its clinical relevance.

**Methods:**

We analyzed consecutive patients who underwent a MitraClip procedure to treat MR between August 2010 and March 2019 in the Heart Failure Network Rhineland registry. RVF was assessed before and after the procedure. Impaired RVF was defined as an RV fractional area change (RVFAC) < 35% or tricuspid annular plane systolic excursion (TAPSE) < 16 mm.

**Results:**

816 eligible patients (77 ± 9 years, 58.5% male) were included in the analysis. Baseline values of RVF were: RVFAC 38.6 (IQR 29.7–46.7) % and TAPSE 17.0 (IQR 14.0–21.0) mm. At a median time of 3 (IQR 2–5) days after the procedure, the RVF remained normal in 34% (*n* = 274), normalized in 17% (*n* = 140), deteriorated in 15% (*n* = 125), and was persistently impaired in 34% (*n* = 277) of patients. The RVF response was significantly associated with a composite outcome of all-cause mortality and hospitalization due to heart failure within a 2-year follow-up. Compared to stable/normal RVF, the adjusted hazard ratios for the outcome were 1.78 (95% CI 1.10–2.86) for normalized RVF, 1.89 (95% CI 1.34–3.15) for deteriorated RVF, and 2.25 (95% CI 1.47–3.44) for persistently impaired RVF. Changes in TAPSE and RVFAC as continuous variables were significantly correlated with the outcome.

**Conclusion:**

An early change in RVF following transcatheter mitral valve repair is predictive of mortality and hospitalization due to heart failure during follow-up.

**Graphic abstract:**

Early response of RVF after MitraClip and its clinical significance. An acute, early change in RVF can be observed following the MitraClip procedure, which is associated with the risk of mortality and hospitalization for HF.

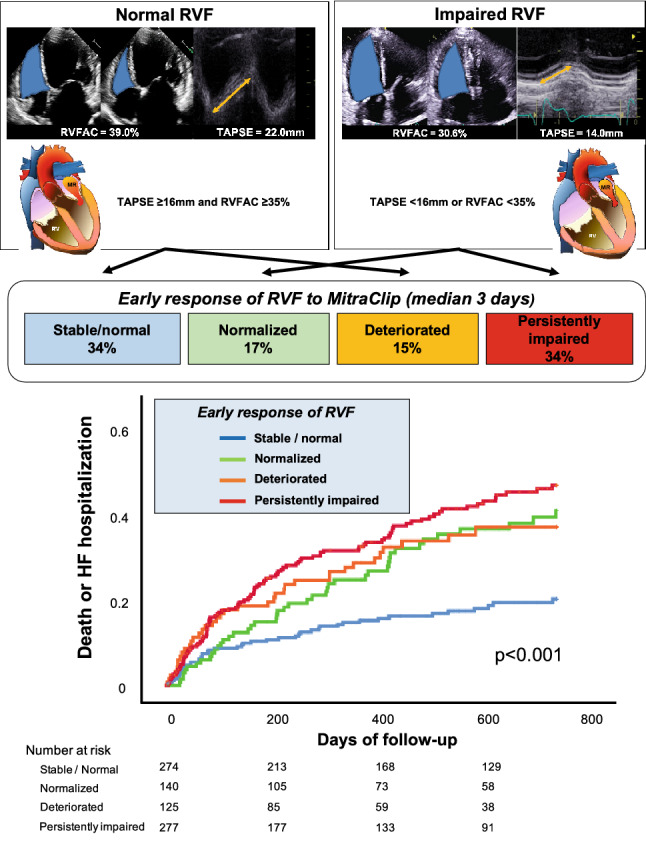

**Supplementary Information:**

The online version contains supplementary material available at 10.1007/s00392-021-01951-7.

## Introduction

Mitral regurgitation (MR) is the most common valvular heart disease [1], which induces pulmonary hypertension and increases RV afterload [2]. Right-ventricular function (RVF) is a pivotal prognostic marker in patients with MR: impaired RVF has been associated with dismal clinical prognosis, as well as an increased risk of mortality and hospitalization due to heart failure (HF) [3].

Treating MR may promote RV unloading and eventually recovery of function, with a potential prognostic benefit. Transcatheter edge-to-edge mitral valve repair using the MitraClip system is a less invasive therapeutic option for patients with MR who are at a high risk of surgical complications [4]. Because RVF is sensitive to both the volume and pressure load, this procedure may offer an acute benefit for RVF. In contrast, an acute deterioration of RVF has also been reported following the MitraClip and is linked to worse clinical outcomes. Nevertheless, data on the early RV response following MitraClip is still lacking. Accordingly, we aimed to investigate the RV response after MitraClip and its impact on clinical outcomes.

## Methods

### Study design and setting

We conducted a retrospective analysis of data from the Heart Failure Network Rhineland registry—a multicenter, prospective, consecutive database that includes information for patients treated at university hospitals in Bonn, Cologne, and Duesseldorf [5]. We reviewed data for patients who underwent MitraClip (Abbott Vascular Inc., Menlo Park, CA) for the treatment of MR between August 2010 and March 2019. All patients had symptomatic MR and were considered ineligible for surgery or at a high risk of surgical complications. After a standard diagnostic work-up, which included transesophageal echocardiography and left-heart catheterization, patients were evaluated for the treatment of MR by an interdisciplinary heart team at the individual centers. In accordance with standard institutional protocols, echocardiographic evaluations were performed at baseline and after the procedure. Patients without baseline and post-procedural echocardiographic data were excluded from the analysis.

This study was approved by the ethics committees of the individual centers and was conducted in accordance with the Declaration of Helsinki. All patients provided written informed consent to participate in the registry.

### Echocardiographic analysis

Echocardiographic assessments were performed in accordance with current guidelines [6, 7]. All measurements were reviewed by two independent cardiologists, who were dedicated to echocardiographic evaluation and blinded to the present study. We assessed the severity of MR as follows: grade 0: none, 1 + : mild, 2 + : moderate, 3 + : moderate-to-severe, and 4 + : severe. RV dysfunction was defined as the RV fractional area change (RVFAC) < 35%, which was calculated as [RV end-diastolic area − RV end-systolic area]/RV end-diastolic area × 100, or tricuspid annular plane systolic excursion (TAPSE) < 16 mm [8]. We examined each parameter of RVF before and after the MitraClip procedure. Patients were divided into four groups according to their acute change in RVF: stable/normal, normalized, deteriorated, or persistently impaired RVF.

### Clinical endpoints

The primary outcome was a composite of all-cause mortality and hospitalization due to HF within 2 years. Outcomes were prospectively assessed during scheduled hospital visits or via telephone interviews with the patients’ general practitioners and families.

### Statistical analysis

Categorical variables are reported as percentages and were compared using Fisher’s exact tests. Continuous variables are reported as the mean ± standard deviation (SD) or as medians and interquartile ranges (IQRs), as appropriate. Analyses of variance (ANOVAs) were used to compare continuous variables across groups. Changes in RVF before and after MitraClip (within a few days) were assessed using paired *t* tests or Wilcoxon signed-rank tests.

The Kaplan–Meier method was used to generate event-free survival curves according to an acute response of RVF. The unadjusted and adjusted Cox proportional hazard models were conducted to estimate the association of the RVF response with outcomes [4, 9]. The models were adjusted for age, sex, coronary artery disease, estimated glomerular filtration rate, New York Heart Association functional class, MR etiology, LV ejection fraction, and TR [4, 9]. We also examined each outcome separately (i.e. mortality, HF hospitalization).

To examine the robustness of our inference, we performed several sensitivity analyses. Cox proportional hazard models were conducted to determine the clinical relevance of the changes in TAPSE (model 1) and in RVFAC (model 2). The models were adjusted for the aforementioned variables and baseline RVF. In addition, we performed a landmark-analysis during two periods, from discharge to 3 months and from 3 months to 2 years. Finally, associations between the response of RVF and clinical outcomes were assessed separately, according to MR etiology or residual MR.

Two-tailed *p* values < 0.05 were considered statistically significant. All statistical analyses were performed using EZR version 1.37 (Saitama Medical Center, Jichi Medical University, Saitama, Japan) and SPSS Statistics version 25.0 (IBM, Armonk, New York).

## Results

### Study population

**Fig. 1:**
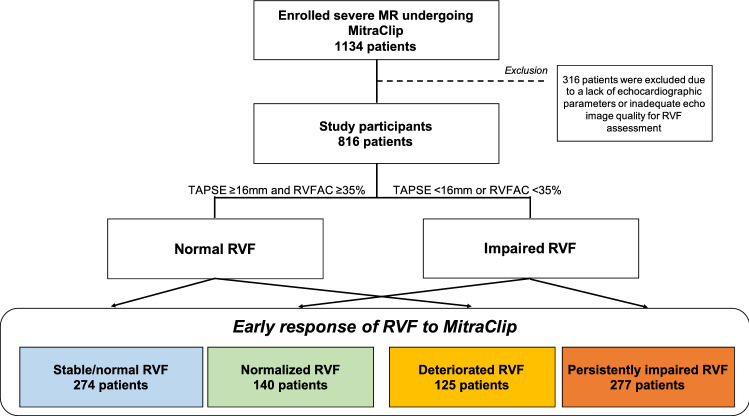
Study population. Baseline and post-procedural RVF data were available for 816 patients. Of these, 399 (49%) patients presented with normal RVF at baseline. Post-procedural echocardiography was performed after a median time of 3 days

Baseline and postprocedural RVF data were available for 816 patients during the study period (Fig. 1). Participants were of advanced age (mean 77 ± 9 years) and predominantly male (58%). The median LV ejection fraction was 44.0 (IQR 31.1–57.6) %, and 60% of patients had secondary MR. The median RVFAC was 38.6 (IQR 29.7–46.7) %, the median TAPSE was 17.0 (IQR 14.0–21.0) mm, and impaired RVF was observed in 417 (51%) patients at baseline. Intra- and inter-class correlations of the RVFAC measurement were 0.907 and 0.831 for baseline and 0.899 and 0.766 for after the procedure. A successful implantation of clips was achieved in 98% of patients.

### Early response of RVF to MitraClip

Post-procedural echocardiography was performed at a median time of 3 (IQR 2–5) days after the procedure. Overall, RVFAC increased significantly (from 38.3 to 40.1%, *p* < 0.001), while there was no significant change in TAPSE (from 17.9 to 18.1 mm, *p* = 0.21). Patients were divided into groups according to their change in RVF. Among the study population, after the procedure, 274 (34%) patients showed stable/normal RVF, 140 (17%) patients showed normalization of RVF, 125 patients (15%) exhibited deterioration of RVF, while 277 (34%) patients exhibited persistently impaired RVF (Fig. 1).

The baseline characteristics of each group are summarized in Table 1. Compared to patients with stable/normal RVF, patients with normalized, acutely impaired, or persistently impaired RVF presented with more advanced cardiovascular disease at baseline as evidenced by having higher levels of N-terminal pro-B-type natriuretic peptide, lower LV ejection fraction, larger LV volume, larger RV area, and more severe tricuspid regurgitation (TR).

**Table 1:** Baseline characteristics according to response of right ventricular function

	Overall (*n* = 816)	Stable/normal (*n* = 274)	Normalized (*n* = 140)	Deteriorated (*n* = 125)	Persistently impaired (*n* = 277)	*p* value
Age, years	77 ± 9	78 ± 8	76 ± 10	78 ± 8	76 ± 9	0.03
Sex male	477 (58.5)	134 (48.9)	76 (54.3)	69 (55.2)	198 (72.6)	< 0.001
Body surface area, m^2^	1.87 ± 0.22	1.86 ± 0.22	1.86 ± 0.22	1.85 ± 0.21	1.90 ± 0.21	0.09
Hypertension	637 (78.1)	213 (77.7)	111 (79.3)	93 (74.4)	220 (79.4)	0.70
Diabetes	242 (29.7)	68 (24.8)	43 (30.7)	33 (26.4)	98 (35.4)	0.04
Atrial fibrillation	545 (66.8)	172 (62.8)	90 (65.2)	90 (72.0)	193 (69.7)	0.20
Coronary artery disease	507 (62.1)	151 (55.3)	91 (65.0)	78 (62.4)	187 (67.5)	0.03
COPD	157 (19.2)	60 (21.9)	24 (17.1)	29 (23.4)	44 (15.9)	0.17
Prior cardiac device implantation^a^	317 (38.9)	78 (28.5)	49 (35.0)	56 (44.8)	134 (48.4)	< 0.001
EuroSCORE, %	18.3 (10.0–30.4)	16.0 (9.0–27.6)	18.4 (10.6–30.1)	19.0 (10.8–29.7)	20.0 (10.5–33.4)	0.02
NYHA class IV, *n* (%)	141 (17.3)	47 (19.1)	22 (18.0)	21 (20.2)	51 (23.2)	0.64
NT-pro-BNP, pg/ml	2884 (1481–6119)	2026 (927–3682)	2706 (1453–5044)	2645 (1614–5767)	4523 (2162–11,913)	< 0.001
Estimated GFR, mL/min/1.73m^2^	46.0 (33.0–61.0)	48.2 (35.9–62.5)	45.8 (35.0–59.0)	49.5 (32.0–62.5)	43.5 (30.4–59.0)	0.11
LV ejection fraction, %	44.0 (31.1–57.6)	53.0 (39.8–60.0)	43.8 (31.7–58.0)	46.3 (33.5–59.1)	34.9 (26.7–50.0)	< 0.001
LV end-diastolic volume index, ml/m^2^	69.8 (53.2–93.3)	64.0 (51.9–81.0)	66.9 (50.0–92.7)	68.2 (52.6–92.0)	81.5 (59.5–104.8)	< 0.001
LV end-systolic volume index, ml/m^2^	36.1 (22.9–61.1)	29.3 (20.2–46.5)	33.0 (20.7–58.99	33.8 (22.9–60.1)	51.5 (29.4–71.2)	< 0.001
Left atrial volume index, ml/m^2^	51.8 (41.1–68.6)	46.8 (36.6–63.3)	52.6 (42.0–73.5)	54.0 (44.7–69.9)	54.6 (44.1–69.0)	0.002
MR ≥ 3 +	687 (84.2)	235 (85.8)	117 (83.6)	96 (76.8)	239 (86.3)	0.09
EROA, cm^2^	0.29 (0.20–0.38)	0.28 (0.20–0.36)	0.35 (0.20–0.35)	0.26 (0.20–0.35)	0.30 (0.21–0.40)	0.06
Secondary MR	487 (60.0)	151 (55.1)	73 (52.1)	81 (64.8)	182 (65.7)	0.01
RVFAC, %	38.6 (29.7–46.7)	45.5 (40.0–50.9)	31.7 (27.3–37.8)	44.3 (39.0–51.2)	29.5 (23.8–34.4)	< 0.001
TAPSE, mm	17.0 (14.0–21.0)	19.0 (17.0–22.0)	15.0 (13.0–19.0)	19.0 (15.0–19.0)	14.0 (12.0–16.0)	< 0.001
RV end-diastolic area index, cm^2^/m^2^	10.6 (8.9–13.1)	9.4 (7.5–11.0)	10.6 (8.9–12.8)	10.8 (9.3–12.9)	12.1 (10.0–15.1)	< 0.001
Right atrial area index, cm^2^/m^2^	12.7 (10.0–16.0)	11.1 (9.0–14.1)	12.1 (10.2–16.5)	13.3 (10.9–16.2)	14.1 (11.6–17.5)	< 0.001
TR ≥ 3 +	168 (20.6)	34 (12.4)	25 (17.9)	34 (27.2)	75 (27.2)	< 0.001
SPAP, mmHg	46.3 (37.0–58.0)	45.0 (35.1–55.0)	48.0 (38.0–59.5)	45.2 (39.0–57.8)	49.0 (37.0–60.0)	0.23

Values are mean ± SD, n (%), or median (25th percentile, 75th percentile)

*COPD* chronic obstructive pulmonary disease, *EROA* effective regurgitant orifice area, *GFR* glomerular filtration rate, *IQR* interquartile range, *LV* left ventricular, *MR* mitral regurgitation, *NYHA* New York Heart Association, *RV* right-ventricular, *RVFAC* right ventricular fractional area change, *SPAP* systolic pulmonary arterial pressure, *TAPSE* tricuspid annular plane systolic excursion, *TR* tricuspid regurgitation

^a^Pacemaker/intracardiac defibrillator/cardiac resynchronization therapy

**Fig. 2:**
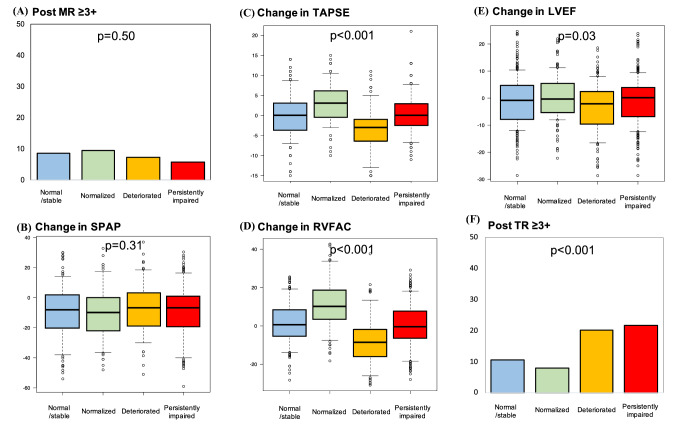
Changes in echocardiographic parameters. A reduction in **a** MR and **b** SPAP was consistently observed among the groups. Changes in **c** TAPSE and **d** RVFAC were correlated. **e** Patients with deteriorated RVF showed a substantial reduction of the LV ejection fraction. **f** Patients with deteriorated or persistently impaired RVF had a higher incidence of TR classified as severe or worse

The echocardiographic parameters following MitraClip are summarized in Supplemental Table 1. A reduction in MR was consistently observed among the groups (*p* = 0.50). As compared to patients with stable/normal or normalized RVF, patients with deteriorated RVF or persistently impaired RVF showed slightly higher pulmonary arterial pressure (*p* = 0.02) and more severe TR (*p* < 0.001) after the procedure. Changes in the main echocardiographic variables are depicted in Fig. 2. We did find a correlation between the change in RVFAC and the change in TAPSE (*r* = 0.14, *p* < 0.001). However, there was no significant difference with regard to a reduction in systolic pulmonary arterial pressure among the groups, and the LV ejection fraction tended to decrease in patients with deteriorated RVF.

### Clinical consequences of the early RVF response

**Fig. 3:**
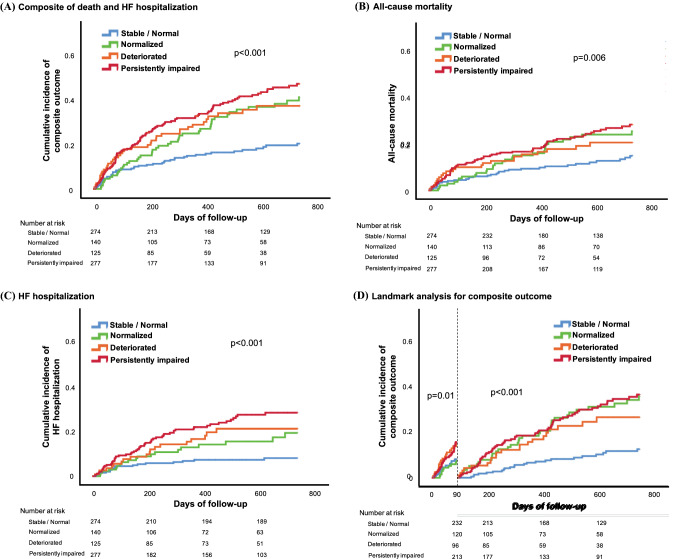
Cumulative incidence of outcomes according to RVF response. Kaplan–Meier curves up to 2-year follow-up for **a** composite outcome of all-cause mortality and HF hospitalization, **b** all-cause mortality, and **c** HF hospitalization, according to the early RVF response. **d** A landmark analysis for the composite outcome during two periods, from discharge to 3 months and from 3 months to 2 years

After a median follow-up of 542 days (IQR 246–897 days), 149 patients had died and 114 patients had been hospitalized due to HF. This means that in total 230 patients (28.2%) experienced the predefined primary endpoint within 2 years after MitraClip. The incidence of the primary outcome differed significantly according to the RVF response (Fig. 3a). Patients with stable/normal RVF had a significantly lower incidence of the primary outcome than patients with acutely deteriorated RVF (20.0% vs. 36.3%, *p* = 0.001). In addition, patients with normalized RVF had a numerically lower incidence of the primary endpoint than patients with persistently impaired RVF (40.1% vs. 46.0%, *p* = 0.13), although the difference did not reach statistical significance at the 2-year follow-up. Similarly, the incidence of all-cause mortality (log-rank *p* = 0.006: Fig. 3b) or HF hospitalization differed significantly (log-rank *p* < 0.001: Fig. 3c) across the groups.

The RVF response was found to be predictive of the occurrence of the primary outcome. The hazard ratios for the composite endpoint vs. stable/normal RVF were 2.05 (1.35–3.10) for normalized RVF, 2.14 (1.38–3.31) for deteriorated RVF, and 2.66 (1.87–3.77) for persistently impaired RVF, all with a *p* value < 0.001 (Table 2). The association of adjusted variables to the composite outcome are presented in Supplemental Table 2. The RVF response was independently associated with the primary endpoint after adjustment for the predefined covariates (Table 2).

**Table 2:** Association between response of RVF and clinical outcomes

	Unadjusted			Adjusted		
	HR	95% CI	*p* value	HR	95% CI	*p* value
Composite outcome						
Stable/normal	Reference			Reference		
Normalized	2.05	1.35–3.10	< 0.001	1.78	1.10–2.86	0.02
Deteriorated	2.14	1.38–3.31	< 0.001	1.89	1.34–3.15	0.01
Persistently impaired	2.66	1.87–3.77	< 0.001	2.25	1.47–3.44	< 0.001
All-cause mortality						
Stable/normal	Reference			Reference		
Normalized	1.78	1.08–2.93	0.02	1.74	1.00–3.04	0.05
Deteriorated	1.60	0.93–2.75	0.09	1.58	0.85–2.92	0.15
Persistently impaired	2.09	1.38–3.18	< 0.001	2.04	1.24–3.38	0.005
HF hospitalization						
Stable/normal	Reference			Reference		
Normalized	2.17	1.14–4.14	0.02	1.77	0.80–3.94	0.16
Deteriorated	2.68	1.41–5.11	0.003	2.38	1.08–5.28	0.03
Persistently impaired	3.63	2.14–6.17	< 0.001	3.06	1.57–5.95	< 0.001

*CI* confidence interval, *HF* heart failure, *HR* hazard ratio, *RVF* right ventricular function

Multivariable models were calculated using the change in TAPSE and that in RVFAC, as shown in Table 3. While a lower TAPSE at baseline was predictive for a worse prognosis, the change in TAPSE was negatively associated with the outcome. Similarly, a decreased RVFAC at baseline was a predictive factor for the outcome, while an increase in RVFAC after MitraClip was associated with a lower risk of the primary composite outcome.

**Table 3:** Cox proportional regression analysis using change in right ventricular function

	Model 1			Model 2		
	Adjusted-HR	95%CI	*p* value	Adjusted-HR	95%CI	*p* value
Change in TAPSE (increase per 1 mm)	0.95	0.91–0.98	0.003			
Baseline TAPSE < 16 mm	1.73	1.19–2.52	0.004			
Change in RVFAC (increase per 10%)				0.83	0.71–0.96	0.01
Baseline RVFAC < 35%				2.10	1.45–3.02	< 0.001
Age (increase per 1 year)	0.97	0.95–0.99	0.007	0.99	0.97–1.01	0.18
Sex male	1.31	0.89–1.91	0.17	1.07	0.74–1.53	0.73
Coronary artery disease	1.57	1.06–2.32	0.02	1.40	0.97–2.03	0.07
Estimated GFR (increase per 1 mL/min/1.73 m^2^)	0.98	0.98–0.99	0.006	0.98	0.98–0.99	0.003
NYHA functional class IV	1.85	1.28–2.68	0.001	1.98	1.40–2.80	< 0.001
Secondary MR	1.13	0.79–1.63	0.50	1.13	0.80–1.60	0.48
LV ejection fraction < 50%	0.84	0.56–1.24	0.38	0.79	0.55–1.13	0.19
TR ≥ 3 +	1.45	0.99–2.11	0.06	1.36	0.95–1.96	0.09

*CI* confidence interval, *GFR* glomerular filtration rate, *HR* hazard ratio, *LV* left ventricular, *MR* mitral regurgitation, *NYHA* New York Heart Association, *RVFAC* right ventricular fractional area change, *TAPSE* tricuspid annular plane systolic excursion, *TR* tricuspid regurgitation

### Landmark analysis

We also evaluated the primary outcome during two distinct time periods, from discharge to 3 months and from 3 months to 2 years (Fig. 3d). Up to 3 months, patients with normalized RVF had a better outcome than those with persistently impaired RVF. In contrast, beyond 3 months, there were no significant differences in the outcome between the normalized, deteriorated, and persistently impaired RVF patients, all of which showed a significantly higher incidence of the primary endpoint compared to patients with stable/normal RVF.

### Subgroup analyses

We analyzed two different subgroups, stratified by MR etiology and LV ejection fraction (Supplemental Fig. 1). The association of the RVF response with the primary composite outcome of mortality and HF hospitalization were consistent across the subgroups by MR etiology or by LV ejection fraction. Cox proportional models are shown in Supplemental Table 3. With the limited sample size, the associations of each RVF response with the primary outcome were consistent across the subgroups.

## Discussion

This is the first study assessing the response of RVF in the acute phase following the MitraClip procedure and its clinical implications for patients with MR. The main findings can be summarized as follows:

1. A change or response in RVF can be observed within a median of 3 days following MitraClip.
2. RVF remained normal in 34%, normalized in 17%, and deteriorated in 15% of patients, while 34% of patients had persistently impaired RVF.
3. The early response in RVF was associated with the primary composite outcome of all-cause mortality and hospitalization due to HF: excess risk persisted after the adjustment (adjusted hazard ratio 1.78 [1.10–2.86] for normalized RVF, 1.89 [1.34–3.15] for deteriorated RVF, and 2.25 [1.47–3.44] for persistently impaired RVF, compared to stable/normal RVF) (Graphical abstract).

### Early response of RVF following MitraClip

Impairment of RVF often coincides with MR because of pulmonary hypertension and increased RV afterload. Decreases in MR can reduce the volume overload of the LA and LV, thereby decreasing RV afterload [10]. Previous cohort studies have reported improvements in RVF 6 months and 1 year after the MitraClip procedure [11–13]. However, given that the RV is sensitive to volume and pressure load, treatment for MR can exert acute effects on RVF in the early postoperative period.

We found that 17% of patients showed a normalization of RVF after MitraClip. Conversely, 15% of the study population showed an acute deterioration of RVF. Such early variation of RVF is novel but consistent with a prior study showing that 20% of patients undergoing MitraClip showed worsening RVF over a median of 4.9-months follow-up [13]. Such deterioration could be a consequence of an iatrogenic atrial septal defect (iASD) [14]. A post-procedural iASD following MitraClip can lead to right-heart failure due to volume overload of the RV and progressive TR [15]. Furthermore, ischemic etiology and reduced LV ejection fraction could be factors associated with increased shunt flow after MitraClip [16]. This relationship can be further underlined by our finding that patients with deteriorated RVF presented with a lower LV ejection fraction and more often had coronary artery disease. Alternatively, the early worsening of RVF could also be attributed to the interplay between LV and RV [13]. In the current study, patients with deteriorated RVF showed a decline in the LV ejection fraction. An increase in LV afterload may occur following a reduction in MR [17], which may, in turn, result in elevated LV filling pressure and thereby exert a negative impact on RVF.

### Clinical consequences of RVF response

Impairment of RVF has been shown to be associated with unfavorable outcomes in the setting of HF. However, conflicting data exist for transcatheter edge-to-edge mitral valve repair [18, 19]. Kaneko et al. reported that baseline impairment of RVF was associated with higher mortality in patients with secondary MR and LVEF < 40% [19], while Godino et al. and Ledwoch et al. have reported that the RVF at baseline did not affect the outcome [13, 18^]^. Our results could contribute to a reconciliation of these conflicting data.

Unloading the RV may promote RV recovery, with a potential prognostic benefit [13^]^. In the current study, acute increases in TAPSE or RVFAC were correlated with a lower risk of the composite outcome at 2 years. Nevertheless, patients with normalized RVF still had a worse outcome compared to patients with stable/normal RVF at 2 years. The worse clinical prognosis of patients with acute normalized RVF after MitraClip might be explained by having more highly impaired kidney function, a higher prevalence of coronary artery disease, and a lower LV ejection fraction. Our findings suggest that baseline comorbidities could attenuate the benefits of RVF improvement over the long-term follow-up.

Rapid worsening of RVF was also found to be detrimental. Acute changes in TAPSE or RVFAC were negatively associated with the risk of outcomes by a Cox regression analysis, independent of baseline RVF. Our findings not only highlight that we should focus on patients who could be at a higher risk for adverse outcomes but also imply that certain adjunctive therapies after transcatheter edge-to-edge mitral valve repair could be beneficial. Closure of the iASD has been linked to reducing the right-heart volume overload [20, 21]. Yet, the implications of iASD deserve some additional consideration. Lurz et al. have recently reported no prognostic benefit of an iASD closure in patients with persistent iASD 1 month after transcatheter mitral valve repair [22]. Further investigation is still needed to identify the subjects who would benefit from an iASD closure, especially with respect to the acute RVF response. Otherwise, given that patients with deteriorated RVF also had more severe TR, transcatheter treatment of their TR might improve the prognosis in this subgroup [23].

Not surprisingly, persistently impaired RVF was associated with the worst outcome. In this subgroup, the RVF impairment may be the consequence of long-lasting RV afterload. Thus, it could be representative of the ‘epiphenomenon’ of intrinsic myocardial damage [24]. Therefore, in these cases, RVF could not be improved by reducing MR by using a MitraClip. The timing of MR interventions should be considered carefully when determining the most appropriate treatment for HF.

### Limitations

Several limitations have to be considered when interpreting our findings. The present study was retrospective in nature, which may have introduced a selection bias. Furthermore, although the present cohort of patients undergoing MitraClip with the assessment of RVF is the largest study of its kind so far, the sample size is still limited. Therefore, the correlation of the primary outcome with RVF response may not have been fully adjusted. Nevertheless, the associations we found were significant across several sensitivity analyses.

## Conclusions

Following transcatheter edge-to-edge mitral valve repair, there can be an acute change in RVF, even after a few days. In this study, the early RVF response was associated with the primary composite outcome of all-cause mortality and hospitalization due to HF. Our findings highlight that RVF should be carefully assessed shortly after transcatheter edge-to-edge mitral valve repair, as well as left-side heart function and MR. Strategies to improve post-procedural hemodynamics need to be explored further.

## Supplementary Information

Below is the link to the electronic supplementary material.

Supplementary file1 (DOCX 18 KB)

Supplementary file2 (DOCX 16 KB)

Supplementary file3 (DOCX 17 KB)

Supplementary file4 Supplemental Figure 1. Subgroup analyses stratified by MR etiology and LV ejection fraction. Shown are Kaplan–Meier curves according to the etiolgy of MR and LV ejection fraction. An association of the RVF response with the primary outcome was consistently observed in patients with (A) primary MR and (B) secondary MR or patients with LV ejection fraction across the subgroups by MR etiology or by (C) LV ejection fraction ≥50% and (D) <50%. Abbreviations: LV, left ventricular; MR, mitral regurgitation; RVF, right-ventricular function (PDF 154 KB)

Supplementary file5 (XLSX 2989 KB)

## Data Availability

All the data underlying this article are available in the article and in its online supplementary material.
