# Supplementary material for: Early response of right-ventricular function to percutaneous mitral valve repair
Source: Clin Res Cardiol. 2021 Oct 20;111(8):859–68. doi: 10.1007/s00392-021-01951-7 (PMC9334433; doi:10.1007/s00392-021-01951-7)
Supplement: Supplementary file 1 — Supplementary file1 (DOCX 18 KB) [file 392_2021_1951_MOESM1_ESM.docx]

**Supplemental Table 1. Echocardiographic parameters at post-procedure evaluation, divided according to acute right ventricular response**

|  | Stable/normal  n = 274 | Normalized  n = 140 | Deteriorated  n = 125 | Persistently impaired  n = 277 | p value |
| --- | --- | --- | --- | --- | --- |
| LV ejection fraction, % | 52.0  (40.0–59.3) | 45.1  (32.1–55.8) | 45.3  (30.1–54.5) | 35.3  (25.5–48.4) | <0.001 |
| LV end-diastolic volume index, ml/m^2^ | 60.1  (43.6–78.1) | 64.9  (47.2–96.9) | 65.0  (47.9–87.2) | 80.4  (58.4–101.2) | <0.001 |
| LV end-systolic volume index, ml/m^2^ | 27.1  (17.8–45.2) | 32.0  (20.3–59.1) | 35.2  (21.8–62.7) | 51.5  (30.9–72.9) | <0.001 |
| Left atrial volume index, ml/m^2^ | 44.2  (36.0–60.7) | 48.3  (37.5–61.1) | 51.1  (40.0–62.5) | 54.7  (43.0–68.4) | <0.001 |
| MR ≥3+ | 23 (8.6) | 13 (9.4) | 9 (7.4) | 16 (5.8) | 0.50 |
| RVFAC, % | 46.7  (41.3–53.7) | 43.6  (38.8–51.8) | 33.9  (29.4–44.1) | 29.5  (24.8–35.9) | <0.001 |
| TAPSE, mm | 21.0  (19.0–25.0) | 19.0  (17.0–21.0) | 15.0  (13.0–17.3) | 14.0  (12.0–16.0) | <0.001 |
| RV end-diastolic area index, cm^2^/m^2^ | 10.2  (8.2–12.2) | 11.0  (9.3–13.4) | 11.6  (9.1–13.2) | 12.8  (10.7–15.1) | <0.001 |
| Right atrial area index, cm^2^/m^2^ | 11.4  (9.3–15.0) | 12.4  (9.7–15.6) | 13.2  (11.0–16.4) | 14.7  (11.9–17.4) | <0.001 |
| Tricuspid regurgitation ≥3+ | 29 (10.7) | 11 (7.9) | 25 (20.2) | 60 (21.8) | <0.001 |
| SPAP, mmHg | 36.0  (29.0–45.0) | 37.0  (30.0–46.0) | 40.0  (31.1–49.0) | 40.0  (32.0–50.0) | 0.02 |

Values are mean±SD, n (%), or median (25th percentile, 75th percentile).

Abbreviations: IQR, interquartile range; MR, mitral regurgitation; RV, right-ventricular; RVFAC, right ventricular fractional area change; SPAP; systolic pulmonary arterial pressure; TAPSE, tricuspid annular plane systolic excursion.
