# Supplementary material for: Early response of right-ventricular function to percutaneous mitral valve repair
Source: Clin Res Cardiol. 2021 Oct 20;111(8):859–68. doi: 10.1007/s00392-021-01951-7 (PMC9334433; doi:10.1007/s00392-021-01951-7)
Supplement: Supplementary file 2 — Supplementary file2 (DOCX 16 KB) [file 392_2021_1951_MOESM2_ESM.docx]

**Supplemental Table 2. Association of adjusting variables to the composite outcome**

|  | Univariable | | | Multivariable | | |
| --- | --- | --- | --- | --- | --- | --- |
|  | **HR** | **95%CI** | **p value** | **HR** | **95%CI** | **p value** |
| Age (increase per 1 year) | 0.99 | 0.97 – 1.00 | 0.06 | 0.98 | 0.96 – 1.00 | 0.05 |
| Sex Male | 1.33 | 1.01 – 1.75 | 0.04 | 1.05 | 0.74 – 1.48 | 0.80 |
| Coronary artery disease | 1.51 | 1.14 – 2.00 | 0.004 | 1.50 | 1.05 – 2.15 | 0.004 |
| Estimated GFR  (increase per 1 mL/min/1.73m^2^) | 0.99 | 0.98 – 0.99 | 0.001 | 0.98 | 0.98 – 0.99 | 0.02 |
| NYHA functional class IV | 2.04 | 1.49 – 2.80 | <0.001 | 1.92 | 1.38 – 2.67 | <0.001 |
| Secondary MR | 1.44 | 1.09 – 1.89 | 0.01 | 1.12 | 0.80 – 1.57 | 0.50 |
| LV ejection fraction < 50% | 1.38 | 1.05 – 1.82 | 0.02 | 0.83 | 0.58 – 1.18 | 0.29 |
| TR ≥3+ | 1.65 | 1.12 – 2.22 | <0.001 | 1.29 | 0.90 – 1.83 | 0.17 |

Abbreviations: CI, confidence interval; GFR, glomerular filtration rate; HR, hazard ratio; LV, left ventricular; MR, mitral regurgitation; NYHA, New York Heart Association.
