# Supplementary material for: Early response of right-ventricular function to percutaneous mitral valve repair
Source: Clin Res Cardiol. 2021 Oct 20;111(8):859–68. doi: 10.1007/s00392-021-01951-7 (PMC9334433; doi:10.1007/s00392-021-01951-7)
Supplement: Supplementary file 3 — Supplementary file3 (DOCX 17 KB) [file 392_2021_1951_MOESM3_ESM.docx]

**Supplemental Table 3. Clinical relevance of acute RVF response to MitraClip**

|  | **Unadjusted** | | | **Adjusted** | | |
| --- | --- | --- | --- | --- | --- | --- |
|  | **HR** | **95%CI** | **p value** | **HR** | **95%CI** | **p value** |
| **Primary MR** | | | | | | |
| **Stable/normal** | Reference | |  | Reference | |  |
| **Normalized** | 3.04 | 1.57 – 5.90 | 0.001 | 3.02 | 1.45 – 6.31 | 0.003 |
| **Deteriorated** | 2.62 | 1.20 – 5.70 | 0.02 | 1.62 | 0.62 – 4.21 | 0.32 |
| **Persistently impaired** | 2.89 | 1.52 – 5.37 | 0.001 | 3.05 | 1.48 – 6.28 | 0.003 |
| **Secondary MR** | | | | | | |
| **Stable/normal** | Reference | |  | Reference | |  |
| **Normalized** | 1.58 | 0.92 – 2.72 | 0.09 | 1.14 | 0.59 – 2.19 | 0.69 |
| **Deteriorated** | 1.82 | 1.08 – 3.08 | 0.03 | 1.80 | 0.97 – 3.35 | 0.06 |
| **Persistently impaired** | 2.38 | 1.56 – 3.63 | <0.001 | 1.70 | 0.99 – 2.91 | 0.05 |
| **LV ejection fraction ≥50%** | | | | | | |
| **Stable/normal** | Reference | |  | Reference | |  |
| **Normalized** | 2.28 | 1.19 – 4.34 | 0.01 | 1.68 | 0.83 – 3.40 | 0.15 |
| **Deteriorated** | 2.12 | 1.09 – 4.15 | 0.03 | 1.81 | 0.83 – 3.93 | 0.14 |
| **Persistently impaired** | 3.29 | 1.85 – 5.84 | <0.001 | 2.94 | 1.55 – 5.56 | <0.001 |
| **LV ejection fraction <50%** | | | | | | |
| **Stable/normal** | Reference | |  | Reference | |  |
| **Normalized** | 1.63 | 0.91 – 2.91 | 0.10 | 1.58 | 0.82 – 3.07 | 0.17 |
| **Deteriorated** | 2.03 | 1.11 – 3.72 | 0.02 | 1.79 | 0.89 – 3.59 | 0.09 |
| **Persistently impaired** | 2.22 | 1.37 – 3.60 | 0.001 | 1.85 | 1.05 – 3.25 | 0.03 |

Abbreviations: CI, confidence interval; HR, hazard ratio; MR, mitral regurgitation; LV, left ventricular.
