# Supplementary figures and images for: Early response of right-ventricular function to percutaneous mitral valve repair
Source: Clin Res Cardiol. 2021 Oct 20;111(8):859–68. doi: 10.1007/s00392-021-01951-7 (PMC9334433; doi:10.1007/s00392-021-01951-7)

(A) Primary MR

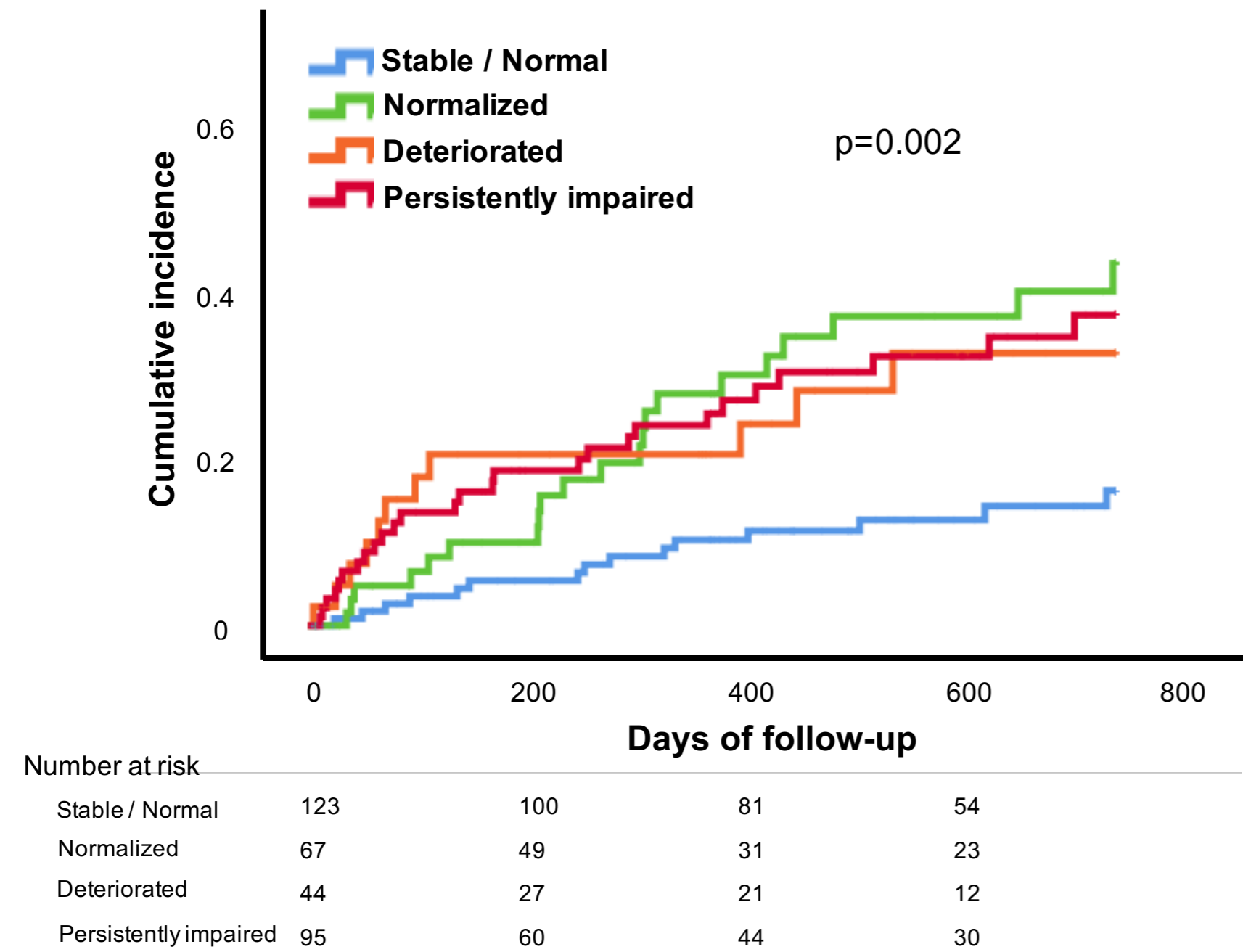

(B) Secondary MR

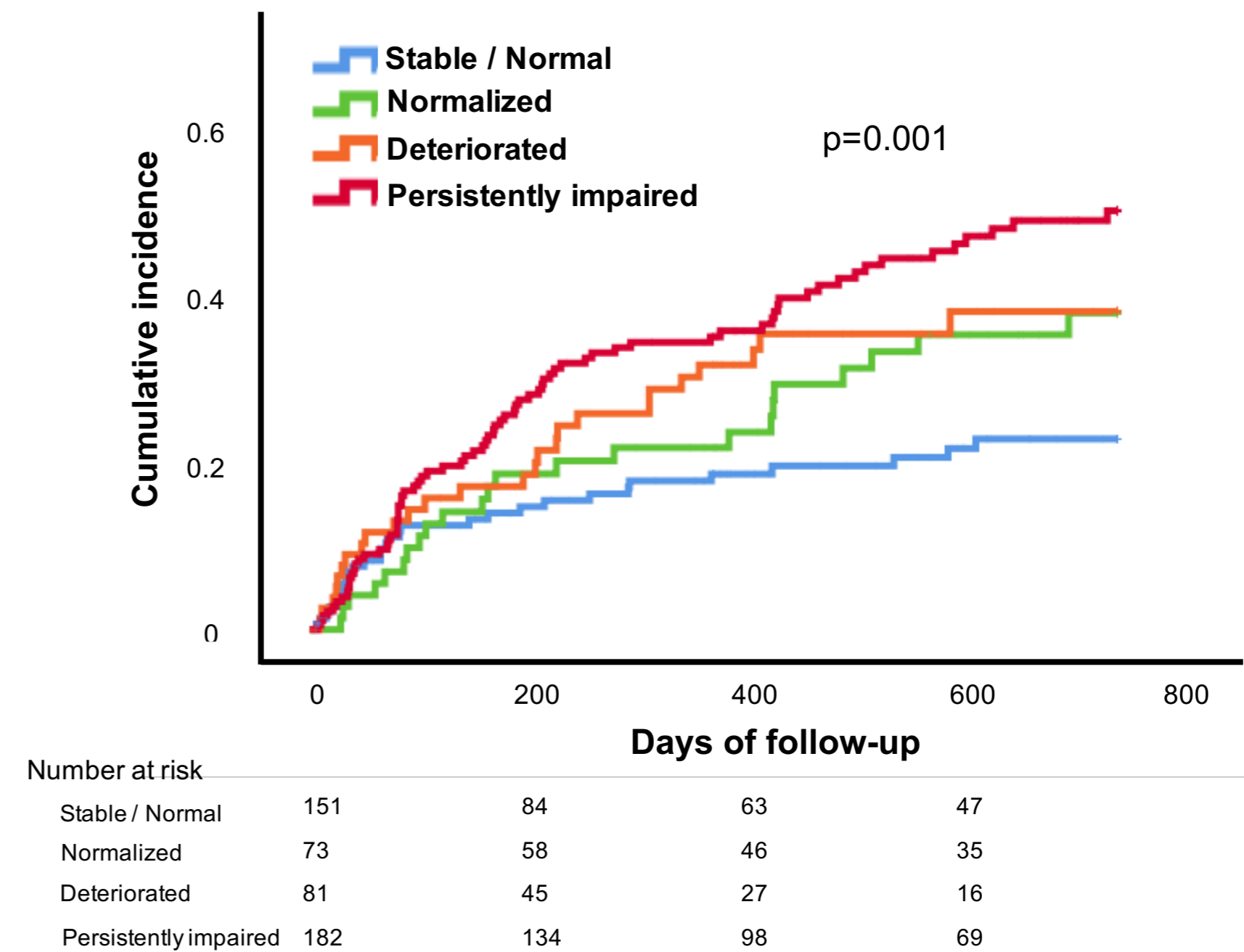

(C) LV ejection fraction ≥50%

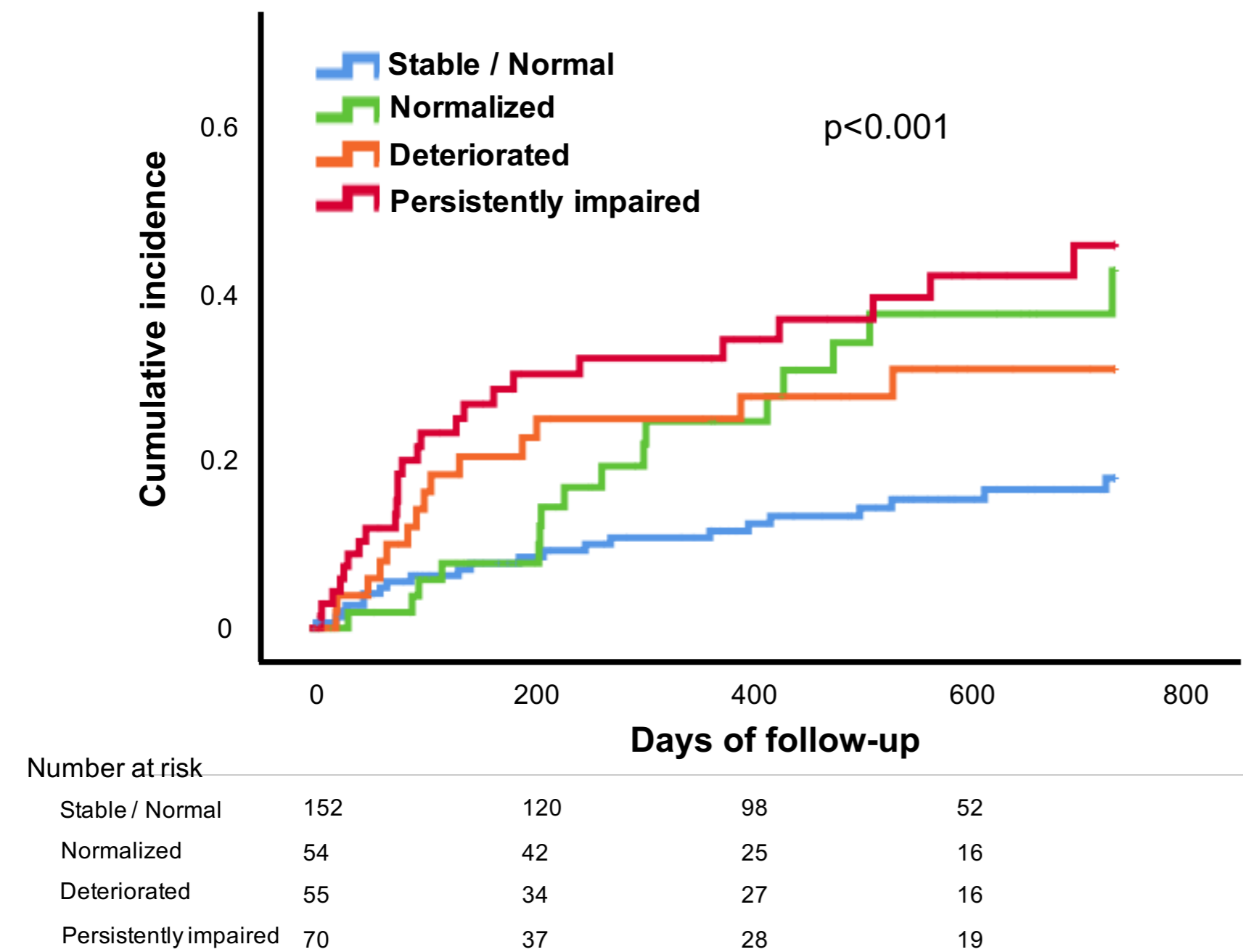

(D) LV ejection fraction <50%

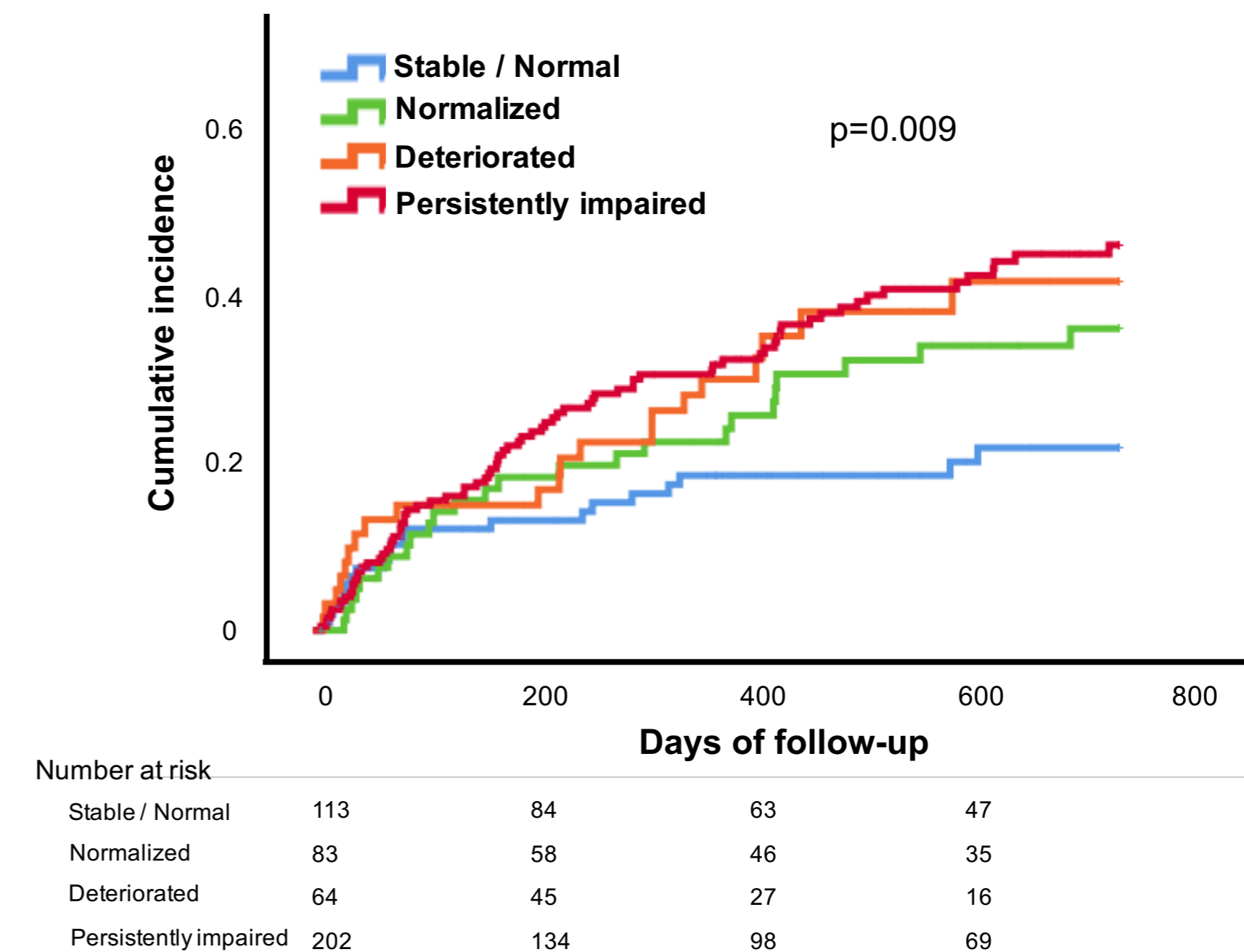

Supplement: Supplementary file 4 — Supplementary file4 Supplemental Figure 1. Subgroup analyses stratified by MR etiology and LV ejection fraction. Shown are Kaplan–Meier curves according to the etiolgy of MR and LV ejection fraction. An association of the RVF response with the primary outcome was consistently observed in patients with (A) primary MR and (B) secondary MR or patients with LV ejection fraction across the subgroups by MR etiology or by (C) LV ejection fraction ≥50% and (D) <50%. Abbreviations: LV, left ventricular; MR, mitral regurgitation; RVF, right-ventricular function (PDF 154 KB) [file 392_2021_1951_MOESM4_ESM.pdf]
